# Supplementary material for: PagMYB205 Negatively Affects Poplar Salt Tolerance through Reactive Oxygen Species Scavenging and Root Vitality Modulation
Source: Int J Mol Sci. 2023 Oct 22;24(20):15437. doi: 10.3390/ijms242015437 (PMC10607357; doi:10.3390/ijms242015437)
Supplement: Supplementary file 1 [file ijms-24-15437-s001.zip › Supplementary Figures.pdf]

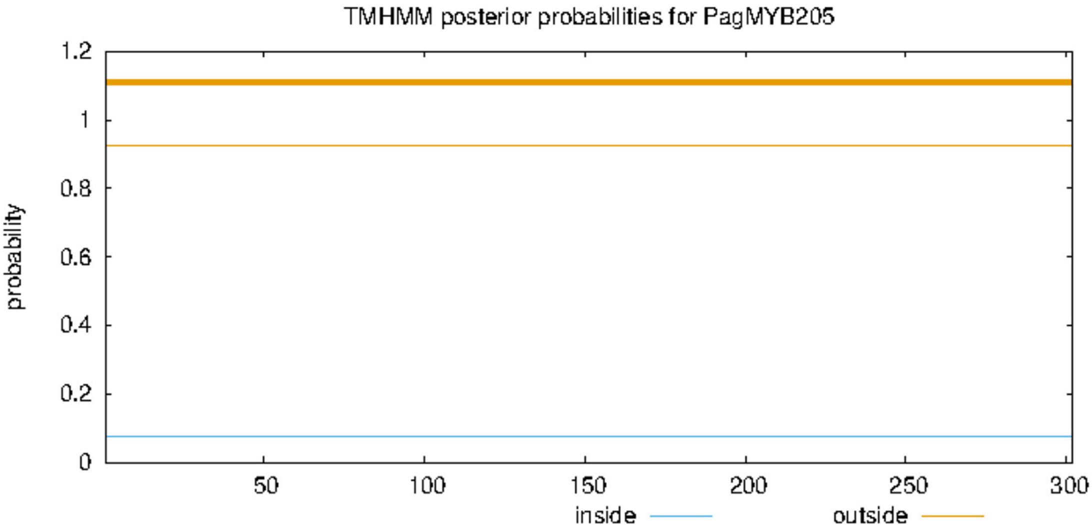

Figure S1 Prediction of protein transmembrane structure.

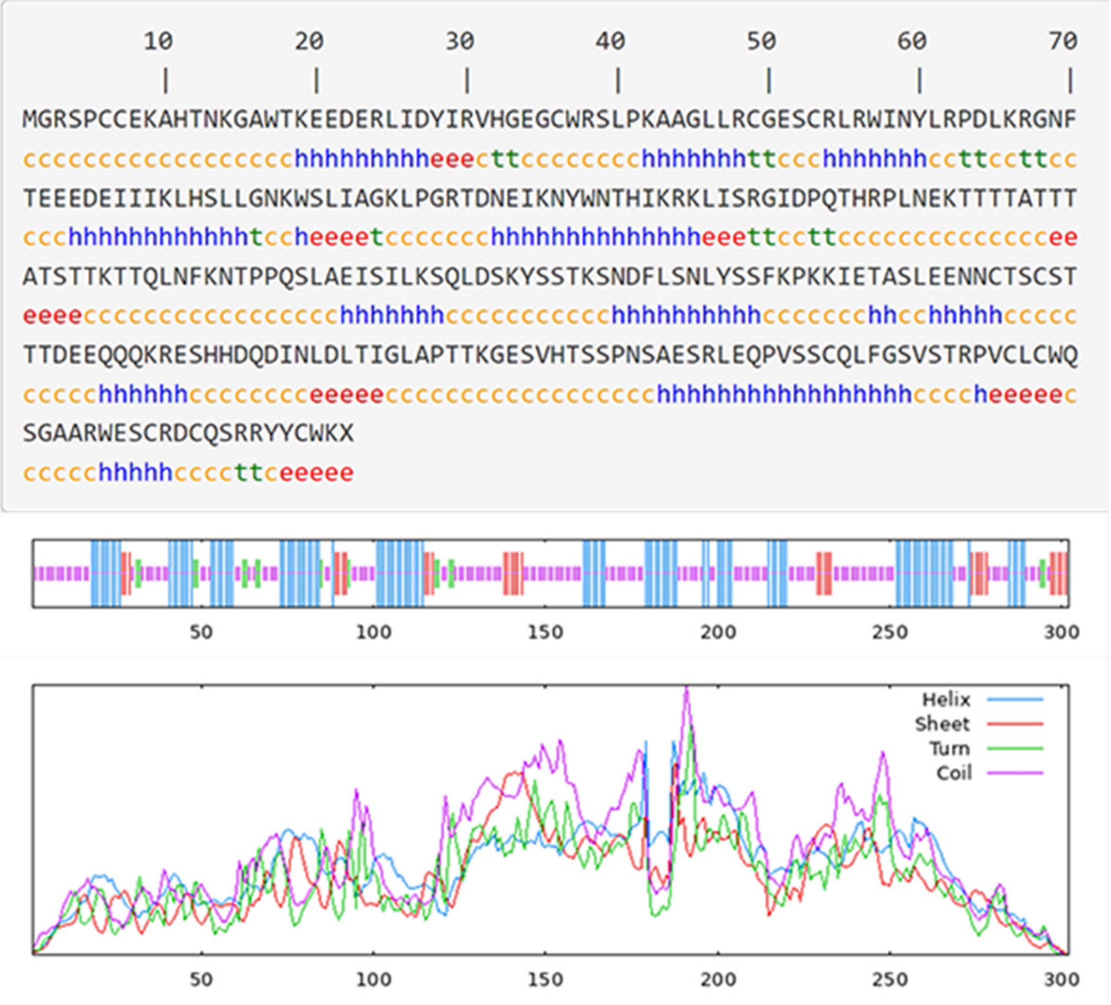

Figure S2 Secondary structure predictive. The blue H stands for α-helix, the red E stands for extended segment, the green T stands for β-angle, and the yellow C stands for irregular curl. The

blue line represents the α-helix, the red line represents the extended segment, the green line represents the β-angle, and the purple line represents the irregular curl.

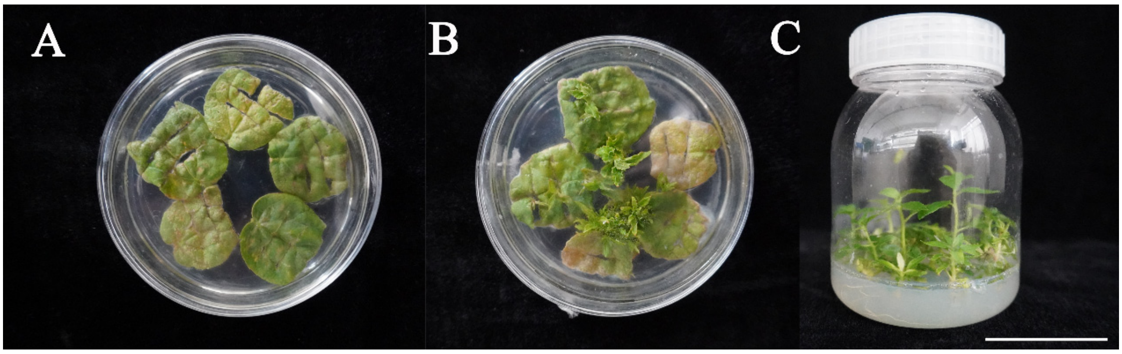

Figure S3 Cultivation of PagMYB205 transgenic poplar. (**A**) Infected 84K poplar leaves were cultured on selective medium. (**B**) The leaves with resistant buds grew on the selective medium. (**C**) Resistant buds grew on selective rooting medium. bar:5cm.

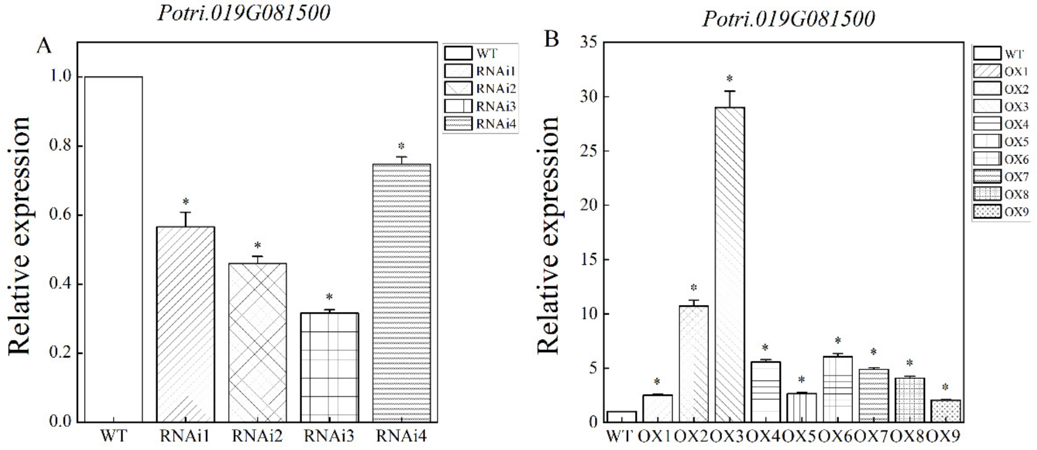

Figure S4 RT-qPCR identification of transgenic poplar. (**A**) Different lines of RNAi-PagMYB205 transgenic poplar. (**B**) Different lines of pBI121-PagMYB205 transgenic poplar. * indicate significant differences among lines ($p < 0.05$).

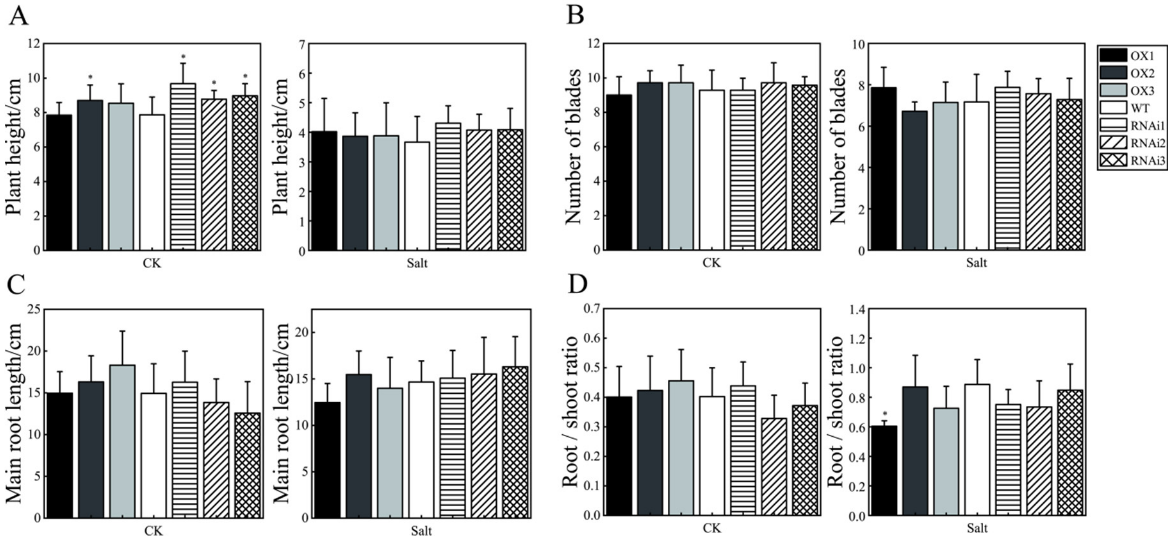

Figure S5 The morphological indexes of PagMYB205 transgenic poplar and non-transgenic poplar under salt treatment and non-salt treatment conditions. (**A**) Plant height. (**B**) Number of blades. (**C**) Main root length. (**D**) Root-shoot ratio. * indicate significant differences among lines ($p < 0.05$). CK: normal conditions; Salt: Salt treatment conditions.

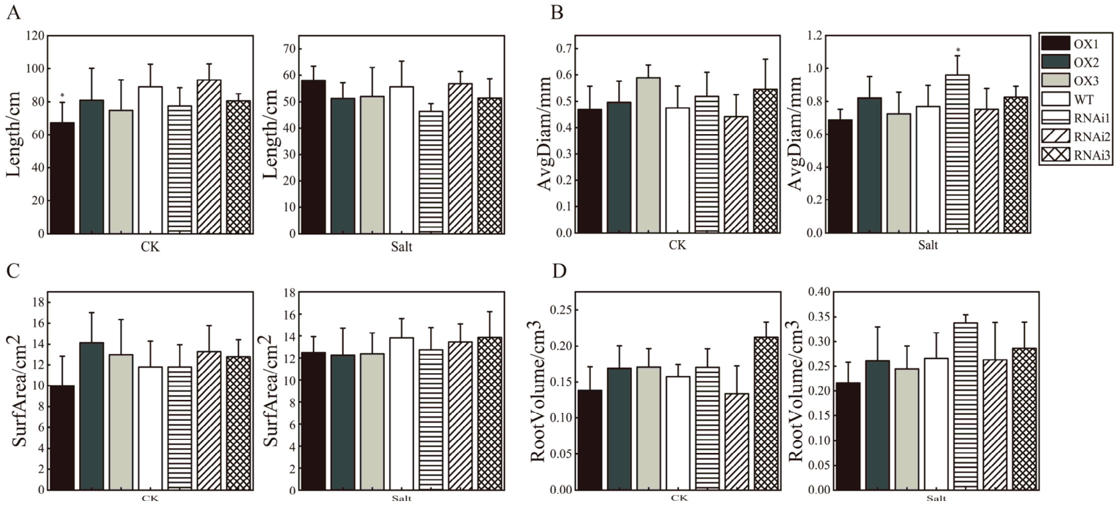

Figure S6 Root morphological data of PagMYB205 transgenic poplar and non-transgenic poplar under salt and non-salt treatment conditions. (**A**) Length. (**B**) AvgDiam. (**C**) SurfArea. (**D**) Root Volume. * indicate significant differences among lines ($p < 0.05$). CK: normal conditions; Salt: Salt treatment conditions.

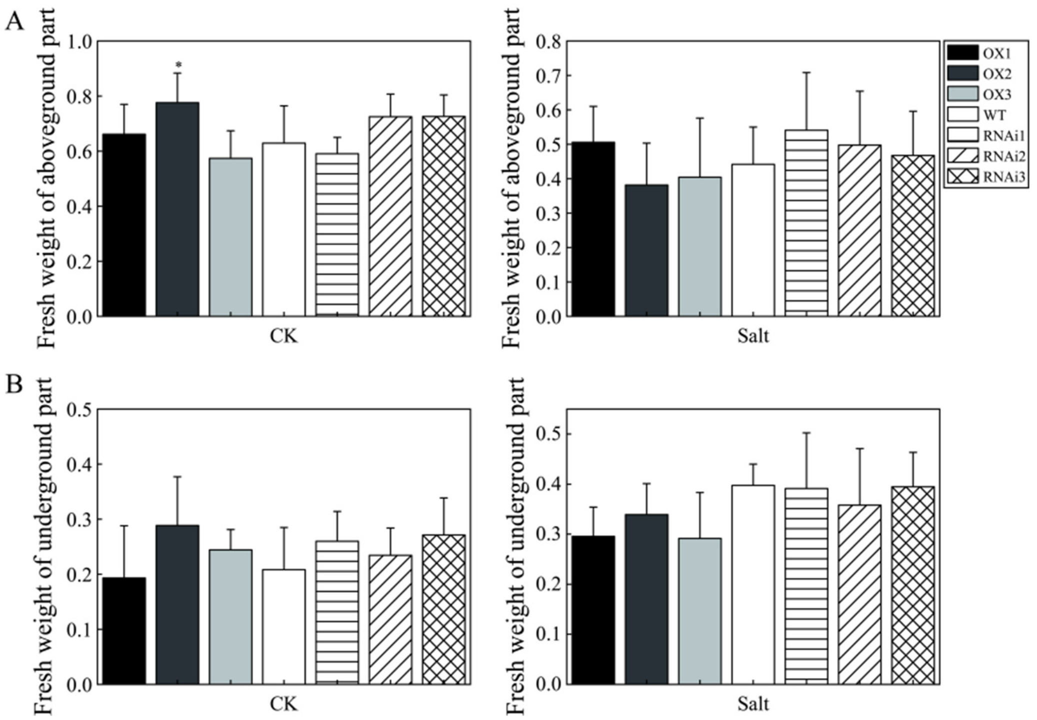

Figure S7 Fresh weight of PagMYB205 transgenic poplar and non-transgenic poplar under salt treatment and non-salt treatment conditions. (**A**) Fresh weight of aboveground part. (**B**) Fresh weight of underground part. * indicate significant differences among lines (p<0.05).CK: normal conditions; Salt: Salt treatment conditions.
