# Supplementary material for: PagMYB205 Negatively Affects Poplar Salt Tolerance through Reactive Oxygen Species Scavenging and Root Vitality Modulation
Source: Int J Mol Sci. 2023 Oct 22;24(20):15437. doi: 10.3390/ijms242015437 (PMC10607357; doi:10.3390/ijms242015437)
Supplement: Supplementary file 1 [file ijms-24-15437-s001.zip › Supplementary Tables.pdf]

Table S1 Physicochemical properties of PagMYB205

Genes	Molecular Weight (Da)	Total number of atoms	Amino Acids	Physicochemical properties						
				Asp + Gl	Arg + Lys	Theoretical pI	Instability index	Aliphatic index	Grand average of hydropathicity (GRAVY)	Estimated half-life (h)
PagMYB205	34166.32	4743	301	36	43	8.73	61.28	64.85	-0.839	30

Table S2 PagMYB205 encodes the amino acid composition of the protein

Amino acid composition	PagMYB205		Amino acid composition	PagMYB205	
Ala (A)	13	(4.3%)	Phe (F)	5	(1.7%)
Arg (R)	20	(6.6%)	Pro (P)	13	(4.3%)
Asn (N)	16	(5.3%)	Ser (S)	33	(11.0%)
Asp (D)	13	(4.3%)	Thr (T)	30	(10.0%)
Cys (C)	13	(4.3%)	Trp (W)	8	(2.7%)
Gln (Q)	12	(4.0%)	Tyr (Y)	7	(2.3%)
Glu (E)	23	(7.6%)	Val (V)	5	(1.7%)
Gly (G)	15	(5.0%)	Pyl (O)	0	(0.0%)
His (H)	8	(2.7%)	Sec (U)	0	(0.0%)
Ile (I)	16	(5.3%)	Asx(B)	0	(0.0%)
Leu (L)	27	(9.0%)	Glx(Z)	0	(0.0%)
Lys (K)	23	(7.6%)	Xaa(X)	0	(0.0%)
Met (M)	1	(0.3%)			

Table S3 Cis-element of *PagMYB205*

Site Name	Function	Number
I-box	part of a light responsive element	1
MRE	MYB binding site involved in light responsiveness	1
TGACG-motif	cis-acting regulatory element involved in the MeJA-responsiveness	3
ARE	cis-acting regulatory element essential for the anaerobic induction	2
Box 4	part of a conserved DNA module involved in light responsiveness	2
CGTCA-motif	cis-acting regulatory element involved in the MeJA-responsiveness	3
TCCC-motif	part of a light responsive element	1
G-Box	cis-acting regulatory element involved in light responsiveness	3
ABRE	cis-acting element involved in the abscisic acid responsiveness	2
3-AF1 binding site	light responsive element	1

RY-element	cis-acting regulatory element involved in seed-specific regulation	3
Gap-box	part of a light responsive element	1
GT1-motif	light responsive element	1

Table S4 Primer sequences

Name	Primers（5′→3′）	Length
PF	AGAAGACGTTCCAACCACG	19 bp
PR	CGGTAAGGATCTGAGCTACAC	21bp
CAM-RNAi-PagMYB205-F1	ATTTGGAGAGGACACGCTCGAGATTGACCCACAAACACACA	41bp
CAM-RNAi-PagMYB205-R1	ACCAAGCTGGGGTACCGAATTCACTCTCCCTCTTTTGTTGT	41bp
CAM-RNAi-PagMYB205-F2	TGGGTTCGAAATCGATAAGCTTACTCTCCCTCTTTTGTTGT	41bp
CAM-RNAi-PagMYB205-R2	CTCATTAAAGCAGGACTCTAGAATTGACCCACAAACACACA	41bp
pBI121-PagMYB205-F	ATGGGTCGCTCACCTTGTTGCG	19bp
pBI121-PagMYB205-R	GATTACAGGGTCAGGTGAAG	21bp
pBI121-F	CCATCGTTGAAGATGCCTCTGC	22bp
pBI121-R	CTCTTCGCTATTACGCCAGCTG	22bp
PagMYB205-BD-F	CCGGAATTCATGGGTCGCTCACCTTGTTG	29bp
PagMYB205-BD-R	ACGCGTCGACCCAACAATAATATCTACGGCTC	32bp
pGBKT7-F	TCATCGAAGAGAGTAGT	17bp
pGBKT7-R	GAGTCACTTTAAAATTTGTATA	22bp
PagMYB205-F	CCGCCACAACCACCGCTACTTC	22 bp
PagMYB205-R	CTTCAAGAGAAGCGGTCTC	19bp
Actin-F	ACCCTCCAATCCAGACACTG	20 bp
Actin-R	TTGCTGACCGTATGAGCAAG	20 bp
